# Comparative chloroplast genome analyses of *Oxytropis* DC. species: new insights into genome evolution and phylogenomic implications

**DOI:** 10.3389/fpls.2025.1645582

**Published:** 2025-08-28

**Authors:** Qin-Qin Li, Yan Niu, Zhi-Ping Zhang, Jun Wen, Chen-Yang Liao

**Affiliations:** ^1^ College of Life Science and Technology, Inner Mongolia Normal University, Hohhot, China; ^2^ Key Laboratory of Biodiversity Conservation and Sustainable Utilization in Mongolian Plateau for College and University of Inner Mongolia Autonomous Region, Hohhot, China; ^3^ College of Computer Science and Technology, Inner Mongolia Normal University, Hohhot, China; ^4^ Department of Botany, National Museum of Natural History, Smithsonian Institution, Washington, DC, United States; ^5^ College of Architecture and Environment, Sichuan University, Chengdu, China

**Keywords:** *Oxytropis*, adaptive evolution, chloroplast genome, comparative analyses, phylogeny

## Abstract

The genus *Oxytropis* DC. comprises about 310 species distributed in Asia, Europe, and North America. Previous studies based on evidences from morphology or a few molecular markers are helpful for understanding the classification and systematic evolution of *Oxytropis*. However, a scarcity of chloroplast genomic resources for *Oxytropis* has hindered the understanding of the genus’s systematic classification and chloroplast genome evolution. Here comparative genomic analyses were conducted on chloroplast genomes of 24 *Oxytropis* species. Chloroplast genomes of *Oxytropis* species showed the triad structure due to the loss of one copy of the IR, with the size range from 121854 bp to 125271 bp. The *Oxytropis* cp genomes encoded a total of 110 genes, including 76 protein-coding genes (PCGs), 30 transfer RNA (tRNA) genes, and four ribosomal RNA (rRNA) genes. It was found that the *atpF* intron, one *clpP* intron, one *rps12* intron, *rpl22* gene, *rps16* gene, and *infA* gene were lost in the *Oxytropis* cp genomes. Seven regions (*5’-rps12-clpP, clpP* intron*, psbM-petN*, *rpl23-trnI-CAU*, *ndhJ-trnF-GAA,trnQ-UUG-accD*, *trnL-UAA-trnT-UGU*) were chosen as potential molecular markers, which will contribute to species identification, population genetics and phylogenetic studies of *Oxytropis.* The phylogenetic relationships among *Oxytropis* species provided some implications for the classification of *Oxytropis*. Congruent with studies based on the morphological evidence, the close relationships between *O. neimonggolica* and *O. diversifolia*, as well as *O. filiformis* and *O. coerulea* were revealed. The results supported the treatment of *O. daqingshanica* as a separate species and refuted the inclusion of *O. daqingshanica* in *O. ochrantha* as conspecific taxa. In addition, it was suggested that *O. chiliophylla* should be considered as a separate species rather than its inclusion in *O. microphylla*. The 16 positively selected genes (*rps3*, *rps4*, *rps7*, *rps11*, *rps12*, *rpl2*, *rpl20*, *rpl32*, *rpoC2, psbC*, *rbcL*, *atpF, clpP*, *accD, ycf1*, *ycf2*) are related to important biological processes for instance self-replication, photosynthesis and metabolite biosynthesis, which may contribute to the adaptation of *Oxytropis* to its habitats. This study will lay a solid foundation for further studies on species identification, taxonomy, and systematic evolution of *Oxytropis*.

## 1 Introduction

As the third largest flowering plant family after Asteraceae Bercht. & J.Presl and Orchidaceae Juss., the Fabaceae Lindl. comprises about 751 genera and 19,500 species worldwide (LPWG (The Legume Phylogeny Working Group), 2013). Combined with a series of diagnostic characteristics, phylogenetic analyses based on *matK* sequences supported the classification system with six monophyletic subfamilies within the Fabaceae, with Papilionoideae DC. (503 genera, ca. 14,000 species) and Caesalpinioideae DC. (148 genera, ca. 4400 species) as the largest two subfamilies, followed by Detarioideae Burmeist. (84 genera, ca. 760 species), Cercidoideae LPWG (12 genera, ca. 335 species), Dialioideae LPWG (17 genera, ca. 85 species), and Duparquetioideae LPWG (one genus, one species) (LPWG (The Legume Phylogeny Working Group), 2017). Phylogenetic analyses based on plastomes and nuclear genes strongly support the classification system of six Fabaceae subfamilies (Zhang et al., 2020; Zhao et al., 2021), which has currently gained widespread acceptance and consensus among scholars. *Oxytropis* DC. belongs to the Astragalean clade under the inverted-repeat-lacking clade (IRLC) of the subfamily Papillonoideae (LPWG (The Legume Phylogeny Working Group), 2017; Zhao et al., 2021; Duan et al., 2024). The genus *Oxytropis* has about 310 species distributed in Asia, Europe, and North America, with a concentrated distribution in Central Asia (Zhang, 1998; Zhu et al., 2010). *Oxytropis* is an important component of the flora in the alpine and arid regions of the Northern Hemisphere temperate zones, and is one of the common groups in alpine, desert, and semi desert regions (Li and Ni, 1985). The *Oxytropis* plants have certain feeding, medicinal, and ornamental value (Kholina et al., 2021a; Sandanov et al., 2023; Wang B. et al., 2024). Due to the extremely similar morphology between *Astragalus* and *Oxytropis*, the *Oxytropis* species were included in *Astragalus* defined by Linnaeus (1753). De Candolle (1802) first separated *Oxytropis* from *Astragalus* based on the characteristics of keel petal shape and pod septum shape. Delimitation of the subgenera and sections were conducted by taxonomist since the establishment of *Oxytropis*, and although there is a certain consensus, different perspectives also exist (e.g., Bunge, 1874; Vasil’chenko et al., 1948; Pavlov, 1961; Zhang, 1998; Zhu et al., 2010). Micromorphological evidence has been applied to the classification of *Oxytropis* and some insights have been gained (Karaman et al., 2009; Ceter et al., 2013; Erkul et al., 2015; Zhao et al., 2022, 2023). With the development of sequencing technology, molecular markers have been used to address the questions on systematic evolution of *Oxytropis*, however, most studies involved a few molecular markers and referred species sampling with limited geographical ranges due to the focus on regional treatments (e.g., Archambault and Strömvik, 2012; Tekpinar et al., 2016a, 2016b; Kholina et al., 2016, 2021a, 2022). In addition, the cp genome sequences of the *Oxytropis* that can be used for study on systematic evolution are still scarce (Su et al., 2019; Liu et al., 2021; Bei et al., 2022; Tavares et al., 2022). Some progress has been achieved in the phylogenetic study of *Oxytropis*, but there is still a long way to uncover the systematic evolutionary questions for this complex taxonomic group with a large number of species, wide distribution, diverse morphology, and a relatively recent diversification history (Shavvon et al., 2017). The lack of effective molecular markers has to some extent hindered the phylogenetic study of *Oxytropis*, thus employing highly variable molecular markers coupled with increased taxon sampling promise advances in the issues on its taxonomy and evolution. The adaptation of *Oxytropis* species to special habitats makes it an excellent model for studying adaptive evolution, which is still an open issue for *Oxytropis*.

Chloroplast (cp) is a vital organelle in green plants, having a crucial role in photosynthesis and a myriad of metabolic activities (Neuhaus and Emes, 2000; Daniell et al., 2016; Wang J. et al., 2024). In angiosperms, the cp genomes are mostly a quadripartite structure: a large single-copy (LSC) region and a small single-copy region (SSC) separated by two inverted repeats (IRs) (Wicke et al., 2011), however, losses of the IR exist in a few angiosperm families, such as Geraniaceae (Guisinger et al., 2011; Ruhlman et al., 2017), Cactaceae (Sanderson et al., 2015), Arecaceae (Barrett et al., 2016), Fabaceae (Choi et al., 2019), Lophopyxidaceae and Putranjivaceae (Jin et al., 2020), and Passifloraceae (Cauz-Santos et al., 2020). Chloroplast genome has been widely used in studies on taxonomy, phylogeny and evolution of angiosperm (e.g., Kan et al., 2024; Li et al., 2024a; Yan et al., 2024; Wang et al., 2025; Yan et al., 2025), due to its own advantages such as uniparental inheritance, small size, lack of recombination, and moderate nucleotide substitution rate (Palmer, 1985; Wicke et al., 2011; Mower and Vickrey, 2018).

Through comparative genomics analyses of the cp genomes of 24 species of *Oxytropis*, this study aims to (1) explore the basic characteristics of *Oxytropis* cp genomes, (2) screen the hotspot regions as potential molecular markers of *Oxytropis*, (3) provide preliminary insights into the current classification of some *Oxytropis* species, and (4) understand the adaptation of *Oxytropis* species to the environment at the molecular level. Our study will lay a solid foundation for future studies on cp genome evolution, species identification, genetic diversity, and systematic evolution of *Oxytropis*.

## 2 Materials and methods

### 2.1 Plant material, DNA extraction and sequencing

Materials for the 19 *Oxytropis* species in the present study were collected during field trips, with the collected *Oxytropis* plants pressed into herbarium specimens, and fresh and tender leaves dried in silica gel without affecting identification. The collected specimens were identified by referring to relevant reference books (e.g., Li and Ni, 1985; Zhang, 1998; Zhu et al., 2010; Zhao et al., 2019), and all the voucher specimens were preserved in the herbarium of the Inner Mongolia Normal University (NMTC) (**Table 1**). Total genomic DNA was isolated from the silica-dried leaves according to the protocol of Doyle and Doyle (1987). The extracted DNA was fragmented by sonication and then used for construction of short-insert library (insert size, 300 bp) by NEBNext^®^ Ultra™ II DNA Library Prep Kit for Illumina^®^. Finally, the pooled libraries were sequenced by the Illumina NovoSeq platform in Novogene (Beijing, China).

**Table 1:** Summary of chloroplast genome features of *Oxytropis* species.

Species	Voucher	GenBank accession	Size (bp)	Number of genes				GC content (%)	References
				Total	Protein-coding	tRNA	rRNA		
*O. aciphylla* Ledeb. 1	Li QQ 20230602057 (NMTC)	PV684027	122411	110	76	30	4	34.2	This article
*O. aciphylla* Ledeb. 2		MW794135	122173	110	76	30	4	34.3	Unpublished
*O. arctobia* Bunge		MT409175	125271	110	76	30	4	34.0	Tavares et al. (2022)
*O. bicolor* Bunge 1	Li QQ 20230520004 (NMTC)	PV684034	122387	110	76	30	4	34.2	This article
*O. bicolor* Bunge 2		MN255323	122461	110	76	30	4	34.2	Su et al. (2019)
*O. ciliata* Turcz.	Li QQ 20230620045 (NMTC)	PV684026	122272	110	76	30	4	34.2	This article
*O. chiliophylla* Royle ex Benth.	Li QQ 20230720015 (NMTC)	PV694277	122480	110	76	30	4	34.2	This article
*O. coerulea* (Pall.) DC.	Li QQ 20230807007 (NMTC)	PV684031	122121	110	76	30	4	34.3	This article
*O. daqingshanica* Y.Z.Zhao & Zong Y. Zhu	Li QQ 20220820044 (NMTC)	PV694279	122181	110	76	30	4	34.3	This article
*O. diversifolia* E. Peter 1	Li QQ HLT001 (NMTC)	PV684028	122012	110	76	30	4	34.3	This article
*O. diversifolia* E. Peter 2		MT780271	122210	110	76	30	4	34.2	Unpublished
*O. falcata* Bunge		OR491708	122781	110	76	30	4	34.3	Unpublished
*O. filiformis* DC.	Li QQ 20230805042 (NMTC)	PV684033	122321	110	76	30	4	34.2	This article
*O. glabra* DC.		MW349014	122094	110	76	30	4	34.3	Liu et al. (2021)
*O. hirta* Bunge	Li QQ 20230805058 (NMTC)	PV684032	122356	110	76	30	4	34.2	This article
*O. holanshanensis* H. C. Fu	Li QQ 20230804029 (NMTC)	PV694276	123621	110	76	30	4	34.1	This article
*O. latibracteata* Jurtzev	Li QQ 20230805031 (NMTC)	PV694278	121854	110	76	30	4	34.3	This article
*O. microphylla* (Pall.) DC.	Li QQ 20230528014 (NMTC)	PV684029	122453	110	76	30	4	34.2	This article
*O. myriophylla* (Pall.) DC.	Li QQ 20220830082 (NMTC)	OR911498	122251	110	76	30	4	34.2	This article
*O. neimonggolica* C. W. Chang & Y. Z. Zhao	Li QQ 20230803032 (NMTC)	PV684030	122195	110	76	30	4	34.2	This article
*O. ochrantha* Turcz.	Li QQ 20220828007 (NMTC)	PV684024	122228	110	76	30	4	34.2	This article
*O. oxyphylla* (Pall.) DC.	Li QQ 20230807023 (NMTC)	PV684036	122284	110	76	30	4	34.2	This article
*O. proboscidea* Bunge	Li QQ 20230718011 (NMTC)	PV694280	122363	110	76	30	4	34.3	This article
*O. racemosa* Turcz.	Li QQ 20230531062 (NMTC)	PV684037	122172	110	76	30	4	34.3	This article
*O. sericopetala* Prain ex C. E. C. Fisch.	Li QQ 20230718021 (NMTC)	PV684035	122405	110	76	30	4	34.3	This article
*O.* sp*lendens* Douglas		MT409174	122318	110	76	30	4	34.2	Tavares et al. (2022)
*O. squammulosa* DC.	Li QQ 20230620004 (NMTC)	PV684025	122352	110	76	30	4	34.3	This article

### 2.2 Chloroplast genome assembly and annotation

Trimomatic v. 0.33 (Bolger et al., 2014) was used to remove adapters in the obtained raw sequencing data. The filtered raw reads for each species were then used to assemble the cp whole genome sequence by NOVOPlasty v. 4.3.1 (Dierckxsens et al., 2017), with the cp genome sequence of *O. bicolor* (GenBank accession no. MN255323) (Su et al., 2019) as the reference and its *rbcL* sequence as the seed. The cp genome of *O. myriophylla* obtained from our previous study (Niu et al., 2024) was used as the reference to conduct annotations of the 19 *Oxytropis* cp genomes in the present study. The brief procedure for annotating the cp genome of *O. myriophylla* was as follows: following the annotation method of Zhang et al. (2022), GeSeq (Tillich et al., 2017) and CPGAVAS2 (Shi et al., 2019) were used to annotate the cp genome of *O. myriophylla*, with the cp genomes of *O. bicolor* (MN255323), *O. arctobia* (MT409175) and *O.* sp*elendens* (MT409174) designated as custom reference genomes. The annotation results obtained from GeSeq and CPGAVAS2 were imported into Geneious Prime (Kearse et al., 2012) to check the intron/exon boundaries and the start and stop codon positions. If necessary, manual corrections were performed to obtain the elaborated annotated cp genome of *O. myriophylla*. The brief workflow for annotations of the 19 *Oxytropis* cp genomes was as follows: in Geneious Prime, MAFFT (Katoh and Standley, 2013) alignment was performed between the cp genome sequence of *O. myriophylla* with the complete annotation information and the cp genome sequence of other *Oxytropis* species. Based on the alignment results, transferring annotations function in Geneious Prime was used for annotation, and the annotation results were manually checked and proofread to finally generate the complete annotated cp genome of other *Oxytropis* species. The cp genome sequences of *Oxytropis* species with annotation information in gb format were imported into OrganellarGenomeDRAW (Greiner et al., 2019) to draw their cp genome circular maps. Moreover, annotation of other cp genomes obtained from GenBank were checked before being used for analysis.

### 2.3 Comparative chloroplast genome analyses

Comparative analysis was conducted on the basic characteristics of cp genome lengths, GC contents, and gene quantities in 27 cp genomes of 24 *Oxytropis* species using Geneious Prime (**Table 1**). The 27 *Oxytropis* cp genomes were aligned in MAUVE ver. 2.4.0 under the progressiveMauve algorithm (Darling et al., 2004, 2010). Due to cp genomes of *O. falcata and O. arctobia* with inversion, the two cp genome sequences were not used in molecular marker identification. The coding and noncoding regions in 25 cp genomes of 22 *Oxytropis* species were extracted by Geneious Prime, and all the homologous sequences were aligned one by one in MAFFT v. 7.490 (Katoh and Standley, 2013). The final aligned homologous sequences in fasta format were imported into DnaSP v. 6.12.03 (Rozas et al., 2017) and their nucleotide variability (Pi) values were calculated, and finally candidate molecular markers were screened based on the Pi values and sequence lengths.

### 2.4 Phylogenetic analyses

To reconstruct the phylogenetic relationships among *Oxytropis* species under the phylogenetic background of the Astragaglean clade, a total of 46 cp genome sequences from 43 species under the IRLC of the subfamily Papilionoideae were selected for phylogenetic analysis based on Zhao et al. (2021) (**Supplementary Table S1**). Considering the phenomena of gene/intron loss and inversion in the cp genomes of the IRLC of Papilionoideae (Jansen et al., 2008), only protein coding genes (PCGs) were selected for phylogenetic tree construction. Seventy-six PCGs were extracted from the cp genomes by Geneious Prime and each PCG was aligned separately using MAFFT v. 7.490 (Katoh and Standley, 2013). Alignments of genes that were not common to all species and genes with significant length differences (*accD*, *clpP*, *psbL*, *ycf1*, and *ycf2*) were removed. Finally, alignments of the remaining 71 PCGs were concatenated to form the phylogenetic dataset. Bayesian inference (BI) and the maximum likelihood (ML) methods were employed to construct the phylogenetic trees. GTR+I+G was recommended as best-fit model by PartitionFinder2 (Lanfear et al., 2017), and MrBayes v. 3.2.7a (Ronquist et al., 2012) was then used to construct BI tree based on the method of Zhang et al. (2022). The ML tree was constructed by RAxML v. 8.2.12 (Stamatakis, 2014) following the method of Li et al. (2024b). Finally, these two phylogenetic trees were visualized using FigTree v. 1.4.4 (Rambaut, 2018), and *Alhagi* sp*arsifolia, Caragana arborescens, Chesneya acaulis, Corethrodendron multijugum*, and *Tibetia liangshanensis* were designated as the outgroup to root the trees.

### 2.5 Adaptive evolution analyses

Based on the results of phylogenetic analyses, 31 cp genomes involving 24 species of *Oxytropis* and its four related taxa (*Carmichaelia australis*, *Lessertia frutescens*, *Phyllolobium chinense*, and *Sphaerophysa salsula*) were selected for selection pressure analyses. The CodeML program in the PAML software package (Yang, 2007) is currently the most widely used bioinformatics tool for selection pressure analyses. EasyCodeML (Gao et al., 2019) can offer a user-friendly graphical interface for executing CodeML. Site models from CodeML were performed in EasyCodeML with the purpose of detecting positive selection sites of PCGs in *Oxytropis* cp genomes. Seventy-six PCGs shared by *Oxytropis* and its related taxa were firstly extracted from the cp genomes using Geneious Prime. MAFFT (Katoh and Standley, 2013) was then employed to perform multiple alignment of each PCG according to its codons and stop codons were manually deleted in the final alignment matrix. The final alignment of each PCG was concatenated into a supermatrix, which was exported into fasta format as an input file for EasyCodeML. The ML tree constructed based on the supermatrix by RAxML v. 8.2.12 (Stamatakis, 2014) was as an input tree in EasyCodeML (**Supplementary Figure S1**). The likelihood ratio test (LRT) was used to detect positive selection sites with four comparison models: M0 vs. M3, M1a vs. M2a, M7 vs. M8, and M8a vs. M8. With LRT threshold *p*<0.05, Bayesian empirical Bayes (BEB) (Yang et al., 2005) or Naïve empirical Bayes (NEB) (Nielsen and Yang, 1998) analysis was adopted to detect positive selection sites with posterior probabilities ≥0.95.

## 3 Results and discussion

### 3.1 Features of *Oxytropis* chloroplast genome

**Figure 1:**
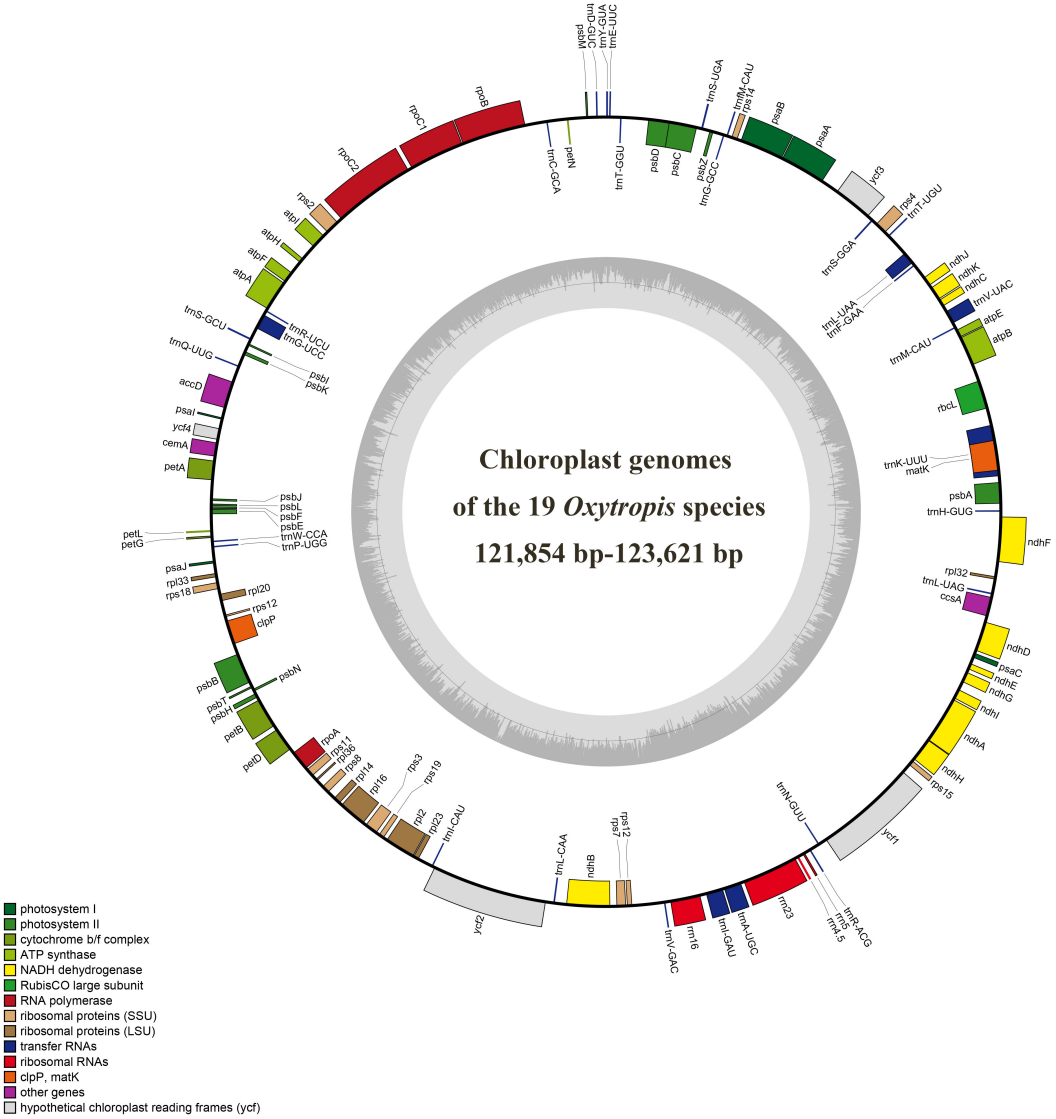
Chloroplast genome map of the 19 *Oxytropis* species generated in this study. Genes outside the circle are transcribed counterclockwise, and those inside are transcribed clockwise.

The size range of cp genomes of 24 *Oxytropis* species was from 121854 bp (*O. latibracteata*) to 125271 bp (*O. Arctobia*) (**Table 1**; **Figure 1**). Compared with the cp genomes of some Papilionoideae taxa such as *Cyamopsis* (Kaila et al., 2017), *Ormosia* (Liu et al., 2019), and *Campylotropis* (Feng et al., 2022) with typical quadripartite structure, the cp genomes of *Oxytropis* species showed the triad structure due to the loss of approximately 25 kb IR. The cp genomes of the IRLC groups in Papillonoideae have lost one IR copy and exhibit the triad structure (Wojciechowski et al., 2004; Jansen et al., 2008). The GC content in the cp genomes of 24 *Oxytropis* species (34.0%–34.3%) was roughly equivalent to that in cp genomes of other IRLC taxa such as *Glycyrrhiza, Astragalus*, and *Galega* (Duan et al., 2020; Su et al., 2021; Feng et al., 2023). The cp genomes of *Oxytropis* species encoded a total of 110 genes, including 76 protein-coding genes (PCGs), 30 transfer RNA (tRNA) genes, and four ribosomal RNA (rRNA) genes (**Tables 1**, **2**). The 110 genes can be classified into four categories according to their functions: 57 genes related to self-replication, 46 associated with photosynthesis, five for other genes, and two genes with unknown function. Moreover, 15 genes contained one intron (*ndhB*, *clpP*, *ndhA*, *rpl16*, *petB*, *rpoC1*, *rpl2*, *petD*, *rps12*, *trnI-GAU*, *trnG-UCC*, *trnL-UAA*, *trnK-UUU*, *trnA-UGC*, *trnV-UAC*), while gene *ycf3* possessed two introns. Gene *rps12* had trans-splicing in the *Oxytropis* cp genome, like in most other angiosperms.

**Table 2:** Genes contained in the *Oxytropis* chloroplast genomes.

Category of genes	Group of genes	Name of genes
Self-replication	Ribosomal RNAs	*rrn4.5*, *rrn5*, *rrn16*, *rrn23*
	Transfer RNAs	*trnA-UGC* ^*^, *trnC-GCA*, *trnD-GUC*, *trnE-UUC*, *trnF-GAA*, *trnfM-CAU*, *trnG-GCC*, *trnG-UCC* ^*^, *trnH-GUG*, *trnI-CAU*, *trnI-GAU* ^*^, *trnK-UUU* ^*^, *trnL-CAA*, *trnL-UAA* ^*^, *trnL-UAG*, *trnM-CAU*, *trnN-GUU*, *trnP-UGG*, *trnQ-UUG*, *trnR-ACG*, *trnR-UCU*, *trnS-GCU*, *trnS-GGA*, *trnS-UGA*, *trnT-GGU*, *trnT-UGU*, *trnV-GAC*, *trnV-UAC* ^*^, *trnW-CCA*, *trnY-GUA*
	Small subunit of ribosome	*rps2*, *rps3*, *rps4*, *rps7*, *rps8*, *rps11*, *rps12* ^*a^, *rps14*, *rps15*, *rps18*, *rps19*
	Large subunit of ribosome	*rpl2* ^*^, *rpl14*, *rpl16* ^*^, *rpl20*, *rpl23*, *rpl32*, *rpl33*, *rpl36*
	DNA dependent RNA polymerase	*rpoA, rpoB, rpoC1* ^*^ *, rpoC2*
Photosynthesis	Subunits of ATP synthase	*atpA*, *atpB*, *atpE*, *atpF*, *atpH*, *atpI*
	Subunits of photosystem I	*psaA*, *psaB*, *psaC*, *psaI*, *psaJ*, ycf3^**^, *ycf4*
	Subunits of photosystem II	*psbA*, *psbB*, *psbC*, *psbD*, *psbE*, *psbF*, *psbH*, *psbI*, *psbJ*, *psbK*, *psbL*, *psbM*, *psbN*, *psbT*, *psbZ*
	Subunits of cytochrome b/f complex	*petA*, *petB* ^*^, *petD* ^*^, *petG*, *petL*, *petN*
	Subunits of NADH-dehydrogenase	*ndhA* ^*^, *ndhB* ^*^, *ndhC*, *ndhD*, *ndhE*, *ndhF*, *ndhG*, *ndhH*, *ndhI*, *ndhJ*, *ndhK*
	Subunit of Rubisco	*rbcL*
Other genes	Subunit of Acetyl-CoA-carboxylase	*accD*
	C-type cytochrome synthesis	*ccsA*
	Envelop membrane protein	*cemA*
	Protease	*clpP* ^*^
	Maturase	*matK*
Unknown function	Conserved open reading frame	*ycf1*, *ycf2*

*Genes containing one intron, **genes containing two introns; ^a^trans-splicing gene.

There are phenomena such as intron loss, gene loss, and inversion in the cp genome evolution of Papilionoideae (Jansen et al., 2008). Our study found the absence of the *atpF* intron, *clpP* intron, and *rps12* intron in the cp genomes of *Oxytropis* and its closely related species. The *atpF* intron is lost in the cp genomes of *Oxytropis*, *Lessertia*, and *Phaerophysa* species, while it is present in *Phyllopium*, *Carmichaelia*, and *Astragalus* species. The absence or presence of the *atpF* intron in the cp genome could be used as a potential molecular marker for distinguishing the morphologically highly similar genera *Oxytropis* and *Astragalus*. Loss of one *clpP* intron was detected in the cp genomes of *Oxytropis* and its closely related taxa, including *Astragalus*, *Carmichaelia, Lessertia, Phyllolobium, and Sphaerophysa* species. The absence of *clpP* intron has also occurred in other IRLC groups of Papillonoideae, for example, one *clpP* intron was lost in *Alhagi*, *Caragana*, and *Vicia* species; and two *clpP* introns were lost in *Tibetia, Corethrodendron, and Glycyrrhiza* species (Jansen et al., 2008; Li et al., 2020; Lee et al., 2021), while the two *clpP* introns are present in other angiosperms genera such as *Uncaria* (Dai et al., 2023), *Alisma* (Lan et al., 2024), and *Argentina* (Li et al., 2024b). The *rps12*-3′-end intron was lost in the cp genomes of *Oxytropis* and its closely related taxa, and the loss of *rps12* intron is a common phenomenon in the IRLC group (Jansen et al., 2008; Lee et al., 2021). Most angiosperm cp genomes contain genes *rpl22*, *rps16*, and *infA*, all of which are lost in the *Oxytropis* cp genomes. The gene *rpl22* is absent in the cp genomes of all legumes (Jansen et al., 2008). We detected that genes *rps16* and *infA* are also not present in the cp genomes of related taxa of the *Oxytropis*, including *Alhagi, Astragalus*, *Caragana, Carmichaelia, Chesneya, Corethrodendron, Lessertia, Phyllolobium, Sphaerophysa*, and *Tibetia* species. Analysis by Mauve showed that among the 27 cp genomes of *Oxytropis*, cp genomes of *O. falcata and O. arctobia* had inversion, while the remaining 25 cp genomes have the same gene order with no obvious reorganization (**Supplementary Figure S2**). The gene order rearrangement did not affect the sequences of any of the involving genes in cp genomes of *O. falcata and O. arctobia*.

### 3.2 Divergence hotspots

**Figure 2:**
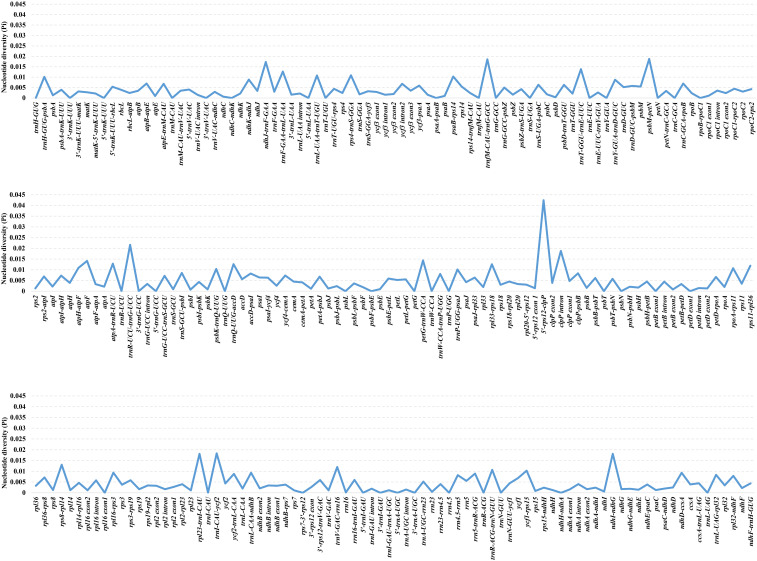
The nucleotide diversity (Pi) values of shared regions in 25 *Oxytropis* chloroplast genomes.

DnaSP v. 6.12.03 (Rozas et al., 2017) was employed to calculate the Pi values of a total of 253 regions of *Oxytropis* cp genome with the aim to screen the highly divergent regions. The Pi values ranged from 0%-4.244%, with a mean value of 0.436%, showing *Oxytropis* cp genomes with a high level of similarity (**Supplementary Table S2**; **Figure 2**). As a whole, 42 regions with Pi =0, 132 regions with 0%<Pi ≤ 0.5%, 50 regions with 0.5%<Pi ≤ 1%, 20 regions with 1%<Pi ≤ 1.5% (*petG-trnW-CCA*, *atpF*, *trnT-GGU-trnE-UUC*, *rps8-rpl14*, *atpA-trnR-UCU, trnF-GAA-trnL-UAA*, *trnQ-UUG-accD*, *rpl33-rps18*, *trnV-GAC-rrn16*, rps11-rpl36, *rps4-trnS-GGA*, *atpH-atpF*, *trnL-UAA-trnT-UGU*, *rpoA-rps11*, *trnR-ACG-trnN-GUU*, *psbK-trnQ-UUG*, *psaB-rps14*, *ycf1-rps15*, *trnH-GUG-psbA*, *trnP-UGG-psaJ*), and nine regions with Pi>1.5% (*5’-rps12-clpP, trnR-UCU-trnG-UCC*, *clpP intron*, *psbM-petN*, *trnfM-CAU-trnG-GCC*, *trnI-CAU-ycf2*, *ndhI-ndhG*, *rpl23-trnI-CAU*, *ndhJ-trnF-GAA*). Among the 29 regions with Pi>1%, 27 regions (excluding *clpP* intron and *atpF*) were located in the intergenic region, indicating that the non-coding regions exhibited higher variation compared to the coding regions, and regions located in the intergenic spacers (IGS) with greater potential for development of molecular markers.

In order to screen molecular markers with potential for development, 19 regions with Pi >1% and alignment lengths >300bp were identified as candidate molecular markers for *Oxytropis*, namely *5’-rps12-clpP, clpP intron*, *psbM-petN*, *ndhI-ndhG*, *rpl23-trnI-CAU*, *ndhJ-trnF-GAA, atpF*, *trnT-GGU-trnE-UUC*, *rps8-rpl14*, *trnQ-UUG-accD*, *rpl33-rps18*, rps11-rpl36, *atpH-atpF*, *trnL-UAA-trnT-UGU*, *trnR-ACG-trnN-GUU*, *psbK-trnQ-UUG*, *ycf1-rps15*, *trnH-GUG-psbA*, and *trnP-UGG-psaJ*. Among these 19 markers, taking into account both Pi value and sequence alignment length, seven regions (*5’-rps12-clpP, clpP intron, psbM-petN*, *rpl23-trnI-CAU*, *ndhJ-trnF-GAA,trnQ-UUG-accD*, *trnL-UAA-trnT-UGU*) were selected as potential molecular markers for *Oxytropis*. The cp molecular markers used in previous phylogenetic studies of *Oxytropis* (Kulshreshtha et al., 2004; Wojciechowski et al., 2004; Artyukova et al., 2011; Tekpinar et al., 2016a, 2016b; Kholina et al., 2016, 2018a, 2018b, 2020, 2021a, 2021b, 2021c, 2022; Chen et al., 2020; Kozyrenko et al., 2020; Sandanov et al., 2023) included *matK*, *rpoC1*, *rpoC2*, *trnL* intron, *trnV* intron, *trnL-trnF*, *trnH-psbA*, *petG-trnP*, and *trnS-trnG.* All other markers except *trnH-psbA* were not among the developed candidate molecular markers for *Oxytropis*, which suggested the significance of developing molecular markers for specific taxonomic groups. Overall, our newly screened potential molecular markers will contribute to species identification, population genetics and phylogenetic studies of *Oxytropis*.

### 3.3 Phylogenetic analyses

**Figure 3:**
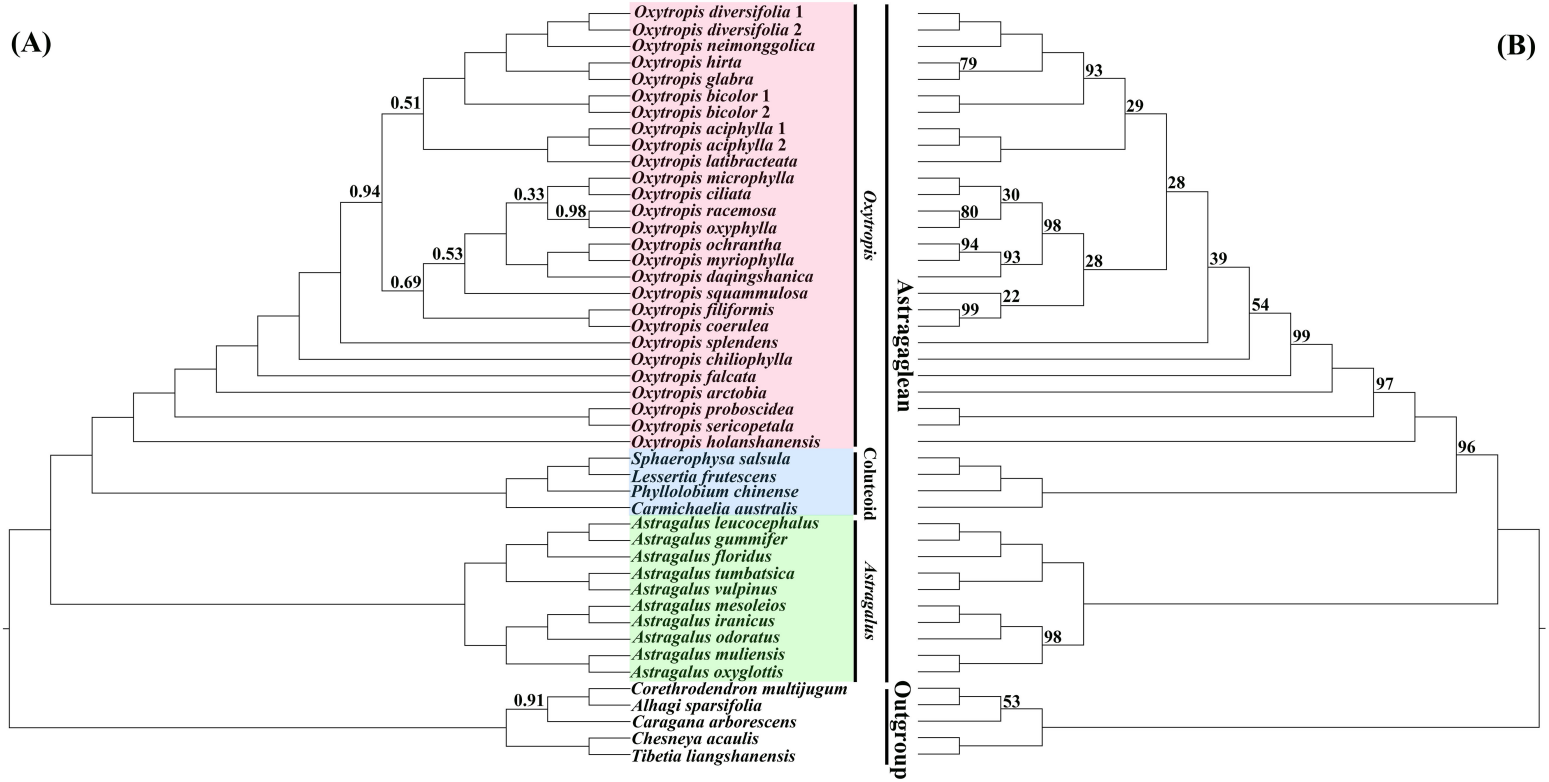
Phylogenetic trees of *Oxytropis* and its related taxa based on the dataset of 71 concatenated protein-coding genes (PCGs) of the chloroplast genomes. **(A)** Bayesian inference (BI) tree, **(B)** maximum likelihood (ML) tree. Values along branches indicate Bayesian posterior probabilities (only PP < 1.00 are shown) and ML bootstrap percentages (only values < 100% are shown), respectively.

Overall, compared to previous phylogenetic studies of *Oxytropis* using several molecular markers obtained with Sanger sequencing (e.g., Kholina et al., 2016; Tekpinar et al., 2016b; Shavvon et al., 2017), relatively high phylogenetic resolution was obtained in our study by utilizing the plastid genome data. Phylogenetic trees inferred from BI and ML analyses were almost identical in topology, and the difference mainly lied in the relative position of *O. squammulosa* versus *O. filiformis* and *O. coerulea* (**Figure 3**; **Supplementary Figures S3, S4**). Both the BI and ML trees showed that the outgroup species were robustly separated from the Astragaglean clade (PP = 1.00, ML BS = 100%). Within the Astragaglean clade, there are three major clades, namely *Oxytropis*, *Astragalus*, and Coluteoid clades. Phylogenetic tree showed that *Oxytropis* species were well clustered together (PP = 1.00, ML BS = 100%), which corroborated the previous studies that *Oxytropis* was monophyletic (e.g., Archambault and Strömvik, 2012; Tekpinar et al., 2016a; Kholina et al., 2016; Shavvon et al., 2017). Consistent with studies of Su et al. (2021); Tian et al. (2021) and Moghaddam et al. (2023) based on cp genome, our result indicated that *Oxytropis* was sister to Coluteoid clade and *Oxytropis*+Coluteoid clade had a sister relationship with *Astragalus.* However, studies of Moghaddam et al. (2016) using ITS, matK and *rpl32-trnL* data and Zhao et al. (2021) based on low-copy nuclear genes revealed that *Oxytropis*+*Astragalus* clade was sisters to Coluteoid clade. The nuclear-cytoplasmic conflict on the phylogenetic position of *Oxytropis* may reflect the complex evolutionary history of this genus. Although the current taxon sampling was still limited, the systematic relationships among *Oxytropis* species showed in our study still provided some insights into the classification of *Oxytropis*. In the phylogenetic trees, *O. neimonggolica* was clustered together with *O. diversifolia* (PP = 1.00, ML BS = 100%), which supported their close relationship based on morphological study (Zhu et al., 2010; Zhao et al., 2019). *Oxytropis ochrantha* and *O. myriophylla* were clustered together and was sister to *O. daqingshanica*, which supported the treatment of *O. daqingshanica* as a separate species (Zhao et al., 2019) and disapproved the inclusion of *O. daqingshanica* in *O. ochrantha* as conspecific taxa (Zhu et al., 2010). *Oxytropis filiformis* was clustered with *O. coerulea* (PP = 1.00, ML BS = 99%), suggesting their close affinity, which was congruent with studies based on the morphological evidences (Zhu et al., 2010; Zhao et al., 2019). *Oxytropis microphylla* and *O. ciliata* grouped together and *O. chiliophylla* was distantly related to these two species, which suggested that *O. chiliophylla* should be considered as a separate species rather than including it in *O. microphylla*. Further taxonomic treatments in *Oxytropis* should be conducted by combining evidence from morphology, anatomy, ecology and palynology.

### 3.4 Adaptive evolution

The p-values of LRTs for compared models M0 vs. M3, M1a vs. M2a, M7 vs. M8, and M8a vs. M8 is below threshold 0.05, suggesting adaptation signatures within *Oxytropis* cp genomes (**Supplementary Tables S3, S4**; **Table 3**). According to the manual of PAML (Yang, 2007), M0 vs. M3 was not suggested as a test of positive selection but as a test of variable ω among sites. In addition, M1a vs. M2a seems to be more stringent compared with M7 vs. M8 which has been confirmed in our results. Therefore, we relied on result under model M8 to discuss positive selection sites in *Oxytropis* cp genomes. Sixteen genes with positive selection sites were detected according to BEB analysis under model M8. The number of positive selection sites in these genes ranged from 1 to 47: nine genes (*rps4*, *psbC*, *rpl20*, *rps12*, *rps11*, *rps3*, *rpl2*, *rps7*, *rpl32*) with one site, two genes (*rpoC2*, *atpF*) having two sites, *clpP* possessing three sites, *rbcL* with five sites, *ycf2* containing eight sites, *accD* with 12 sites, and *ycf1* harboring the largest number of sites. According to their functional category, nine genes (*rps3*, *rps4*, *rps7*, *rps11*, *rps12*, *rpl2*, *rpl20*, *rpl32*, *rpoC2*) were associated with self-replication, three genes (*psbC*, *rbcL*, *atpF*) were responsible for photosynthesis, genes *clpP* and *accD* belonged to other genes, and genes *ycf1* and *ycf2* are functionally unknown.

**Table 3:** Positively selected sites (*: P>95%; **: P>99%) detected in the *Oxytropis* chloroplast genomes in comparisons of M7 vs. M8 and M8a vs. M8 under Bayes empirical Bayes (BEB) analysis.

Gene	Positive selected sites	Pr value(ω>1)	Number of sites
*rbcL*	907 A/1091 A/1142 S/1303 E/1333 E	0.985*/0.988*/0.988*/0.959*/1.000**	5
*rps4*	2615 E	0.985*	1
*psbC*	4929 A	0.999**	1
*rpoC2*	7690 T/7830 R	0.968*/0.963*	2
*atpF*	9257 Q/9297 -	0.986*/0.978*	2
*accD*	9923 S/9968 D/10002 Y/10088 D/10089 E/10090 -/10092 -/10098 -/10106 -/10120 T/10129 E/10143 V	0.996**/0.952*/0.968*/0.996**/0.979*/0.969*/0.961*/0.953*/0.971*/0.960*/0.965*/0.980*	12
*rpl20*	11836 L	0.952*	1
*rps12*	12017 -	0.967*	1
*clpP*	12031 V/12093 D/12155 G	0.986*/0.997**/0.995**	3
*rps11*	13670 A	0.967*	1
*rps3*	14240 K	0.990*	1
*rpl2*	14612 L	0.974*	1
*ycf2*	14838 Q/15489 L/15715 H/15790 F/15952 S/16027 Q/16074 R/16172 Q	0.990**/0.963*/0.992**/0.995**/0.962*/0.957*/0.988*/0.965*	8
*rps7*	17335 Q	0.958*	1
*ycf1*	17495 W/17683 H/17690 V/17693 S/17716 N/17747 -/17751 H/17760 Y/17775 L/17797 N/18085 S/18087 V/18088 Q/18110 Y/18111 S/18115 K/18116 P/18120 Y/18136 F/18141 Q/18142 D/18145 I/18176 F/18202 L/18205 Y/18329 T/18364 K/18366 K/18375 N/18376 V/18377 K/18399 F/18489 Y/18612 L/18974 D/19011 -/19033 S/19037 F/19042 G/19050 D/19051 W/19052 A/19055 S/19075 Y/19103 R/19150 R/19155 T	0.967*/0.965*/0.999**/0.982*/0.989*/0.963*/0.996**/0.998**/0.985*/0.981*/1.000**/0.991**/0.998**/0.967*/0.996**/1.000**/0.980*/0.998**/0.996**/0.992**/0.990*/0.999**/0.992**/0.984*/0.981*/0.999**/0.998**/0.986*/0.998**/1.000**/0.984*/0.964*/0.992**/0.977*/0.976*/0.979*/0.954*/0.968*/0.951*/0.992**/0.998**/0.982*/0.984*/1.000**/0.986*/1.000**/0.997**	47
*rpl32*	21547 T	0.980*	1

Amino acids refer to sequence of *O. racemosa*.

The adaptive evolution of these 16 genes may help *Oxytropis* species adapt to their habitats. Among them, *rps3*, *rps4*, rps7, *rps11*, *rps12*, *rpl2*, *rpl20*, and *rpl32* encoded ribosomal subunit proteins. Chloroplast ribosomal proteins are essential for cp ribosome assembly, which plays an important role in plant survival, acclimation and adaptation (Schmid et al., 2024). DNA dependent RNA polymerase subunit beta’’ encoded by *rpoC2*, is one of the components of the core of plastid-encoded polymerase (PEP) which acts as the major transcription machinery of mature chloroplasts (Zhelyazkova et al., 2012; Kindgren and Strand, 2015). The 43-kDa chlorophyll a-binding protein (CP43) encoded by *psbC*, together with CP47 encoded by *psbB*, binds chlorophyll, as an inner light-harvesting complex of photosystem II (PSII) (Landi and Guidi, 2022). The large subunit of Rubisco was encoded by *rbcL* (Wicke et al., 2011). Rubisco mediates the fixation of inorganic carbon from CO2 into organic compounds during photosynthesis (Wilson and Hayer-Hartl, 2018). In most lineages of terrestrial land plants, *rbcL* is under positive selection (Kapralov and Filatov, 2007). ATP synthase CF0 B subunit encoded by *atpF* is one of the important constituents of chloroplast ATP synthase, which using the proton gradient produces ATP that is indispensable for photosynthesis and plant growth (Hahn et al., 2018; Yamamoto et al., 2023). Gene *clpP* in chloroplast is essential for plant development, with an indispensable function for cell viability (Shikanai et al., 2001; Kuroda and Maliga, 2003). The chloroplast gene *clpP* together with a nuclear multi gene family encodes the Clp protease that degrades damaged proteins during environmental stresses (Clarke, 1999; Adam and Clarke, 2002). The key enzyme acetyl-CoA carboxylase (ACCase) regulates *de novo* synthesis of fatty acids in plants (Rawsthorne, 2002). The *accD* gene encodes one of the four subunits of ACCase, which is essential for cell viability, leaf development, and seed development (Madoka et al., 2002; Kode et al., 2005; Caroca et al., 2021). Products encoded by essential genes *ycf1* and *ycf2* of higher plants are essential for plant cell survival (Drescher et al., 2000).

Previous work suggested that *Oxytropis arctobia accD* gene was under positive selection, which might be related to its adaptation to the cold environment in the Arctic (Tavares et al., 2022). Positively selected genes in *Oxytropis* detected in our study were also found under positive selection in some other Fabaceae genera. For example, *rpl2, rpoC2 and accD* were under positive selection in *Pueraria* (Zhou et al., 2023), and so were *rps11*, *clpP*, *accD* and *ycf1* in *Astragalus* (Moghaddam et al., 2023), *rps4*, *rpl32*, *accD* and *ycf2* in *Pterocarpus* (Hong et al., 2020), *rps4*, *rps7*, *rpl32* and *clpP* in *Vicia* (Li et al., 2020), *rps7*, *rpl20*, *atpF*, *ycf1* and *ycf2* in *Caragana* (Cui et al., 2024), and *rps3*, *rps12*, *rpoC2*, *psbC*, *rbcL*, *clpP*, *accD*, *ycf1* and *ycf2* in *Dalbergia* (Li et al., 2022). *Oxytropis* species spread in temperate and cold regions of the Northern Hemisphere in Asia, Europe, and North America, usually thriving in harsh environments such as the Arctic areas and alpine ecosystems (Zhu et al., 2010; Archambault and Strömvik, 2012; Kholina et al., 2016; Tavares et al., 2022). *Oxytropis* species grow in various habitats such as mountains, steppes, prairies, meadows, deserts, semi deserts, forest-steppes, and forests (Zhu et al., 2010; Sandanov et al., 2023; Welsh, 2023). The origin of *Oxytropis* was dated to about 5.6 million years ago, with 95% highest posterior density intervals ranging from 3.61 to 8.07 Ma, which coincides with climate modifications around the Miocene-Pliocene boundary (Shavvon et al., 2017). It was inferred that *Oxytropis* experienced a recent rapid radiation based on its recent age estimates, short interior branch on gene tree, little genetic differences, and diverse morphology and ecological habitats (Shavvon et al., 2017). The 16 positively selected genes in the *Oxytropis* cp genome are related to important biological processes for instance self-replication, photosynthesis and metabolite biosynthesis, which may contribute to the adaptation of *Oxytropis* to diverse habitats, especially under extreme arid and cold conditions. The adaptation of *Oxytropis* to diverse habitats may have to some extent promoted the rapid diversification of *Oxytropis* in its relatively recent evolutionary history.

## 4 Conclusion

In this study, comparative analysis of cp genomes of 24 *Oxytropis* species revealed that their cp genomes exhibited a triad structure, and the cp genome size, GC content, and gene content were conserved. Seven highly divergent regions (*5’-rps12-clpP, clpP* intron*, psbM-petN*, *rpl23-trnI-CAU*, *ndhJ-trnF-GAA,trnQ-UUG-accD*, *trnL-UAA-trnT-UGU*) identified in this study may be potentially utilized as high-resolution DNA barcodes, which will facilitate species identification and phylogenetic and phylogeographic studies of *Oxytropis*. Phylogenetic analysis based on the cp genome sequences supported the monophyly of *Oxytropis* and provided some new insights into the classification of *Oxytropis*. The results indicated that the cp genome can be utilized as an informative molecular marker for enhancing our understandings of evolutionary diversification in *Oxytropis*. Sixteen protein-coding genes (*rps3*, *rps4*, *rps7*, *rps11*, *rps12*, *rpl2*, *rpl20*, *rpl32*, *rpoC2, psbC*, *rbcL*, *atpF, clpP*, *accD, ycf1*, *ycf2*) showed evidence for positive selection, which may contribute to the adaptation of *Oxytropis* to its diverse habitats. Overall, our study improved the understanding of cp genome features, phylogenetic relationships, and adaptive evolution in *Oxytropis*. Employing single-copy nuclear genes coupled with more detailed taxon sampling will facilitate future work on the phylogeny, biogeography, and adaptive evolution of *Oxytropis*.

## Data Availability

The datasets presented in this study can be found in online repositories. The names of the repository/repositories and accession number(s) can be found below: GenBank of NCBI (https://www.ncbi.nlm.nih.gov/genbank/), PV684024-PV684037, and PV694276-PV694280.
