# Supplementary material for: Comparative chloroplast genome analyses of Oxytropis DC. species: new insights into genome evolution and phylogenomic implications
Source: Front Plant Sci. 2025 Aug 28;16:1645582. doi: 10.3389/fpls.2025.1645582 (PMC12423452; doi:10.3389/fpls.2025.1645582)
Supplement: Supplementary file 1 [file DataSheet1.zip › Supplementary Material/Captions for the Supplementary Material files.docx]

Supplementary Material

Comparative chloroplast genome analyses of *Oxytropis* DC. species: new insights into genome evolution and phylogenomic implications

Qin-Qin Li†*, Yan Niu†, Zhi-Ping Zhang†, Jun Wen, Chen-Yang Liao*

**†**These authors have contributed equally to this work

*** Correspondence:** Qin-Qin Li: liqq@imnu.edu.cn; Chen-Yang Liao: chenyangliao@scu.edu.cn

**Supplementary Figure 1**. Maximum likelihood (ML) tree of *Oxytropis* and related taxa based on the concatenated dataset of 76 chloroplast protein coding genes (PCGs) of the chloroplast genomes. Values along branches indicate ML bootstrap percentages (only values < 100 % are shown).

**Supplementary Figure 2.** MAUVE alignment of cp genomes of 24 *Oxytropis* species, with *O. aciphylla* 1 as the reference.

**Supplementary Figure 3.** Bayesian inference (BI) tree of *Oxytropis* and its related taxa based on the dataset of 71 concatenated protein-coding genes (PCGs) of the chloroplast genomes. Values along branches indicate Bayesian posterior probabilities (only PP < 1.00 are shown).

**Supplementary Figure 4.** Maximum likelihood (ML) tree of *Oxytropis* and its related taxa based on the dataset of 71 concatenated protein-coding genes (PCGs) of the chloroplast genomes. Values along branches indicate ML bootstrap percentages (only values < 100 % are shown).

**Supplementary Table 1**. All of the 46 chloroplast genome sequences of *Oxytropis* and its related taxa used for phylogenetic analyses.

**Supplementary Table 2**. The nucleotide diversity (Pi) values of 253 homologous loci in 25 cp genomes of *Oxytropis*.

**Supplementary Table 3.** Positively selected sites (*: P>95%; **: P>99%) detected in the *Oxytropis* chloroplast genomes in comparison of M0 vs. M3 under Naïve empirical Bayes (NEB) analysis. Amino acids refer to sequence of *O. racemosa*.

**Supplementary Table 4**. Positively selected sites (*: P>95%; **: P>99%) detected in the *Oxytropis* chloroplast genomes in comparison of M1a vs. M2a under Bayes empirical Bayes (BEB) analysis. Amino acids refer to sequence of *O. racemosa*.
