# Supplementary figures and images for: Comparative chloroplast genome analyses of Oxytropis DC. species: new insights into genome evolution and phylogenomic implications
Source: Front Plant Sci. 2025 Aug 28;16:1645582. doi: 10.3389/fpls.2025.1645582 (PMC12423452; doi:10.3389/fpls.2025.1645582)

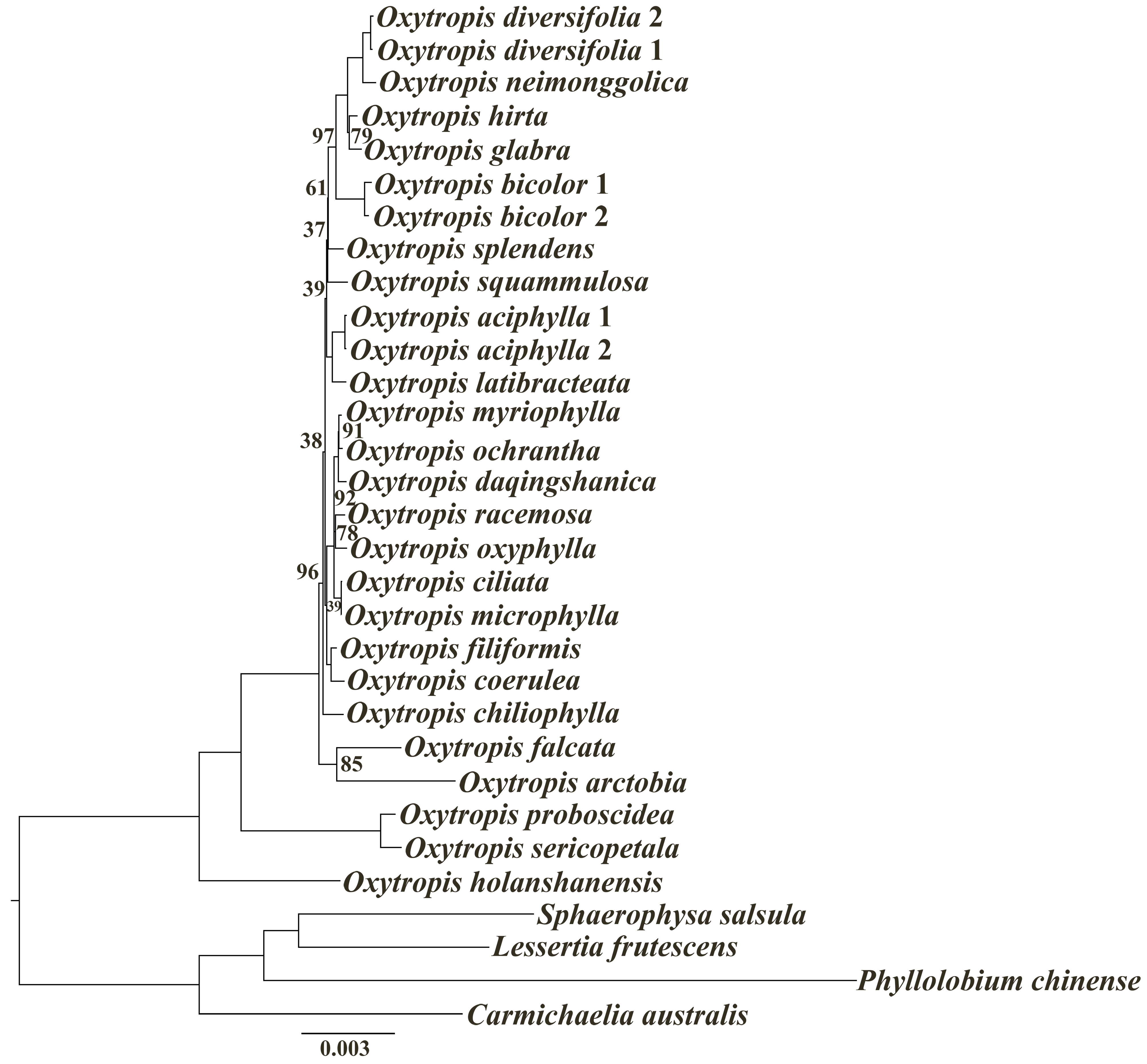

Supplement: Supplementary file 1 [file DataSheet1.zip › Supplementary Material/Figure S1.jpg]

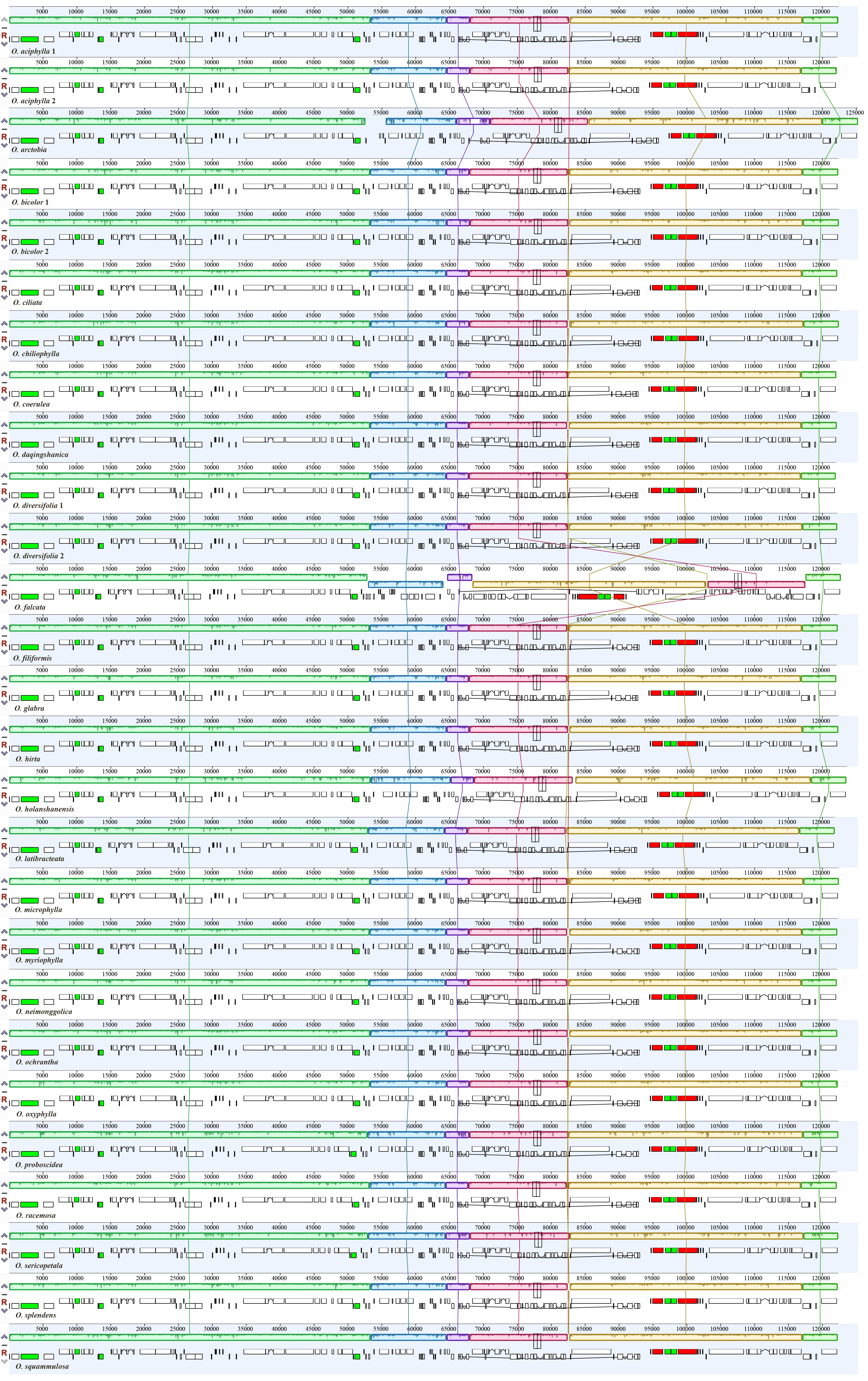

Supplement: Supplementary file 1 [file DataSheet1.zip › Supplementary Material/Figure S2.jpg]
